# Supplementary material for: DMRforPairs: identifying Differentially Methylated Regions between unique samples using array based methylation profiles
Source: BMC Bioinformatics. 2014 May 15;15:141. doi: 10.1186/1471-2105-15-141 (PMC4046028; doi:10.1186/1471-2105-15-141)
Supplement: Additional file 2 — DMRforPairs output for the comparison of A431-MCF7 and NA17018-NA17105. Please start from the HTML files in each folder. Available via the BMC Bioinformatics website. [file 1471-2105-15-141-S2.zip › 1394847754114233_MOESM2_ESM/A431_MCF7/figures/15307.pdf]

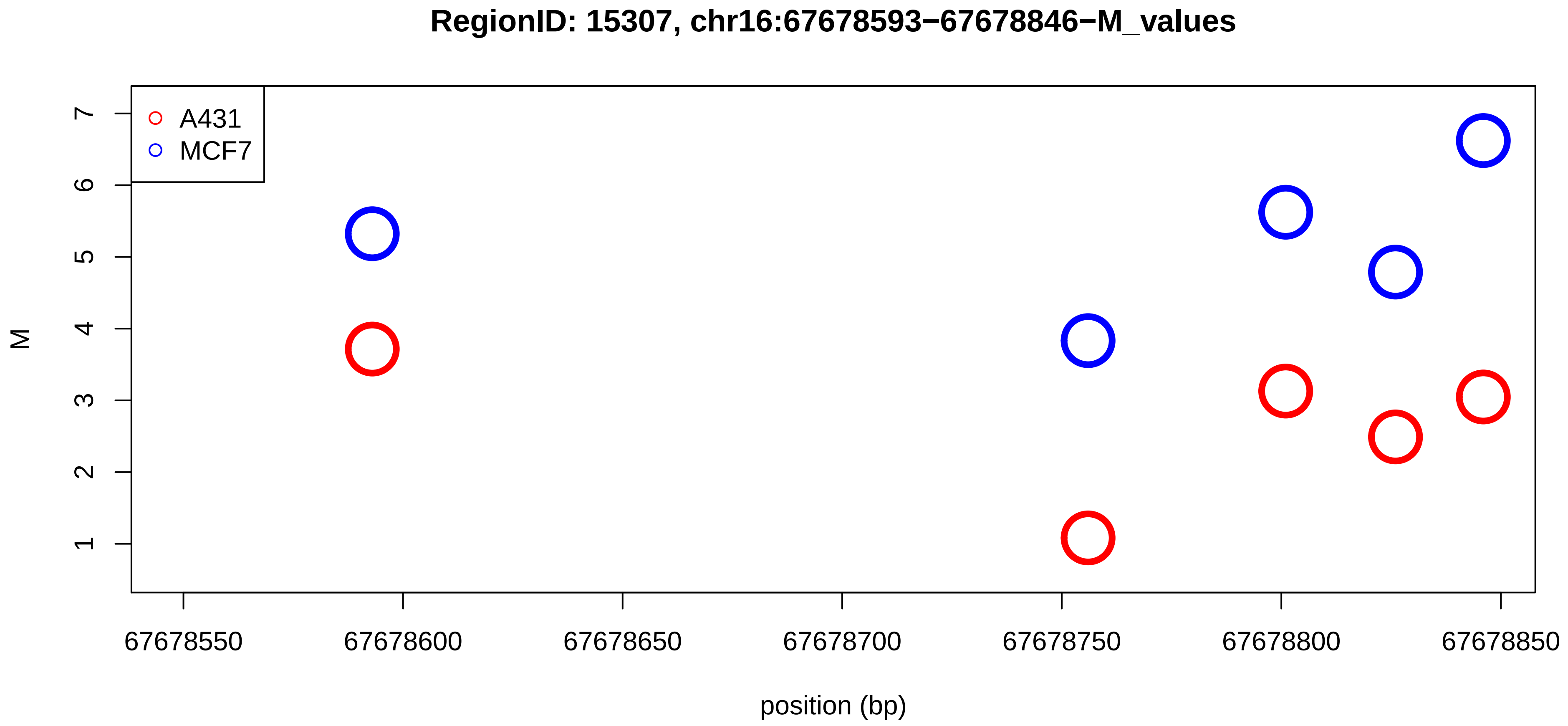
RegionID: 15307, chr16:67678593−67678846−M_values
A431
MCF7
M
1
2
3
4
5
6
7
67678550
67678600
67678650
67678700
67678750
67678800
67678850
position (bp)

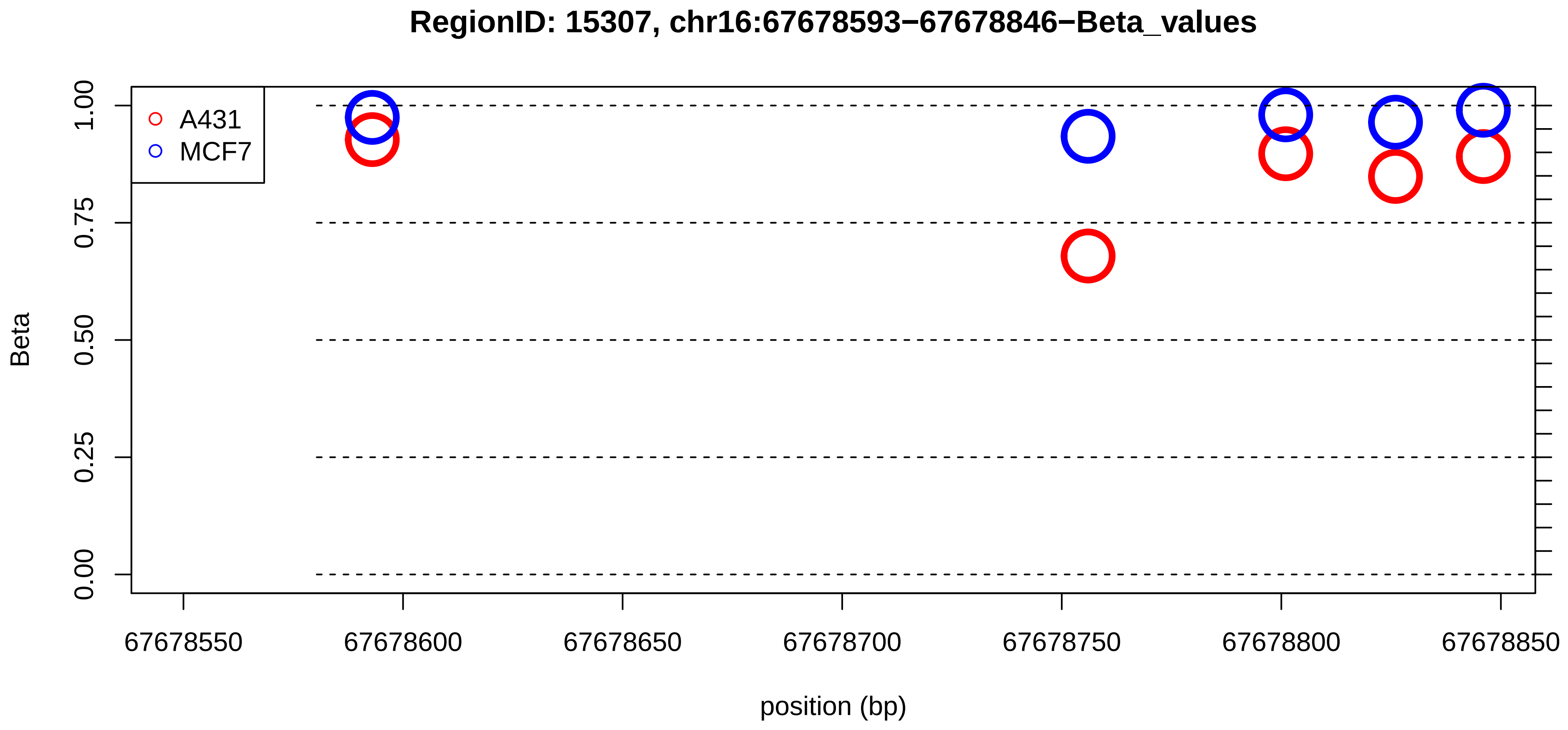
RegionID: 15307, chr16:67678593−67678846−Beta_values
A431
MCF7
Beta
0.00
0.25
0.50
0.75
1.00
67678550
67678600
67678650
67678700
67678750
67678800
67678850
position (bp)

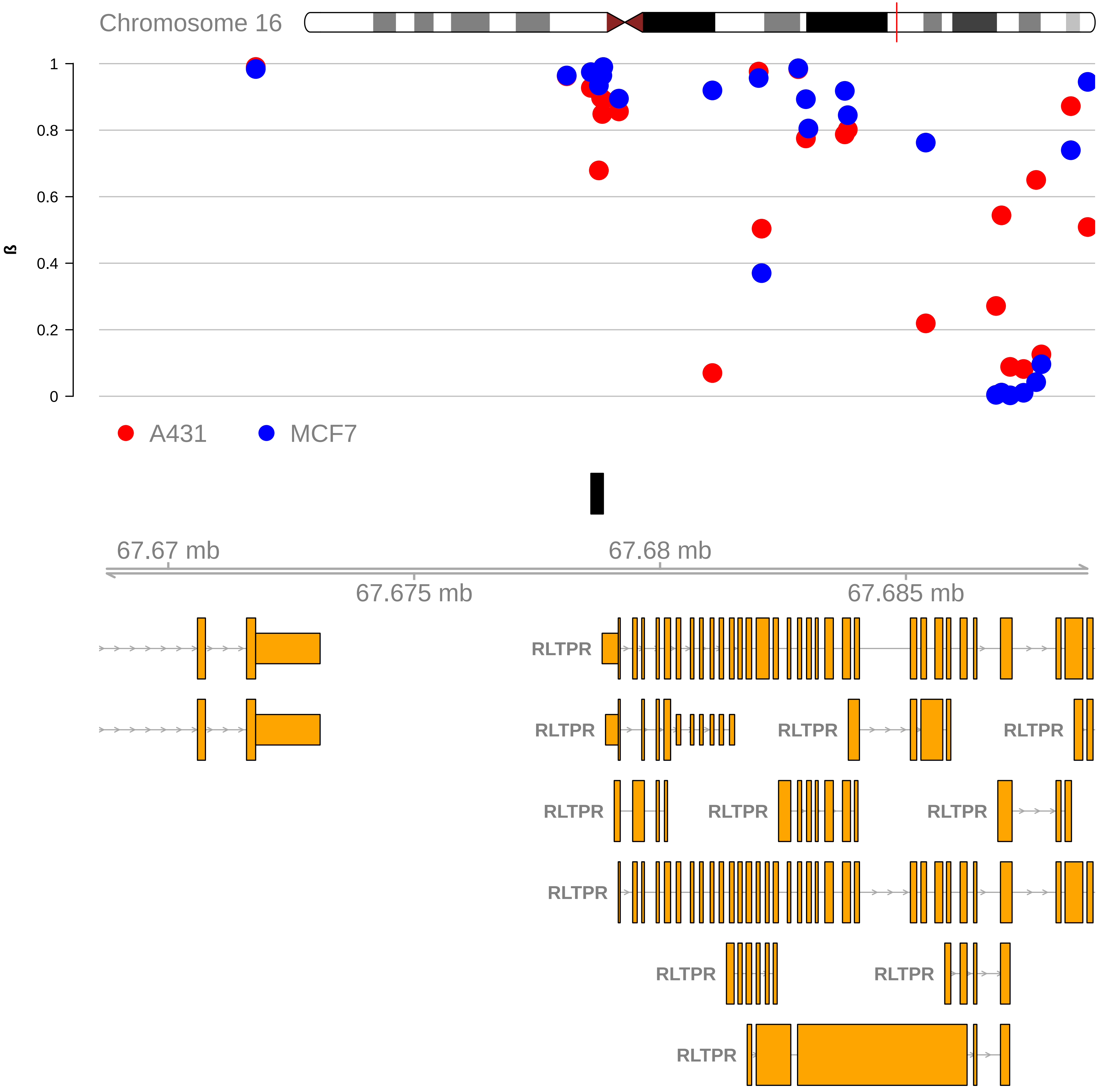
Chromosome 16
1
0.8
0.6
0.4
0.2
0
ß
A431
MCF7
67.67 mb
67.68 mb
67.675 mb
67.685 mb
RLTPR
RLTPR
RLTPR
RLTPR
RLTPR
RLTPR
RLTPR
RLTPR
RLTPR
RLTPR
RLTPR
RLTPR
